# A long-term follow-up study of labor market marginalization in psychiatric patients with and without personality disorder

**DOI:** 10.48101/ujms.v128.9014

**Published:** 2023-07-31

**Authors:** Hanna Spangenberg, Mia Ramklint, Adriana Ramirez

**Affiliations:** Department of Medical Sciences, Uppsala University, Uppsala, Sweden

**Keywords:** Personality disorder, young adulthood, social functioning, labor market marginalization, vocational functioning

## Abstract

**Background:**

Personality disorders (PDs) in adulthood are considered stable over time and are likely to have lasting psychosocial impact on the affected individual, including in areas like vocational functioning. The aim of this study was to study labor market marginalization (LMM) and receipt of social welfare benefits during 13 years from age 18 to 25 years in a sample of former psychiatric patients with and without PD.

**Methods:**

This study followed-up 186 former psychiatric patients who were thoroughly assessed in 2002–2004, including for PD, and compared them with controls. Participants were divided into three groups: former patients with PD, without PD, and a matched control group from the general population. Register data on employment, sick leave absence, disability pensioning, education, days of psychiatric care, income, and receipt of social welfare benefits in 2003–2016 were collected.

**Results:**

Former patients had more days of unemployment, sick leave absence, and disability pensioning and received more social welfare benefits than controls during the study period. Differences between patients with and without PD were smaller than expected, but significant as regards receipt of social welfare benefits. PD also had an effect on income at age 30 years.

**Conclusions:**

Early onset of psychiatric disorders impairs vocational functioning up to 13 years after diagnosis, and most in those with PD.

## Introduction

Personality disorders (PDs) are associated with a wide range of functional impairments affecting psychosocial functioning, including in the occupational setting (1, 2). The impact of PD on work life has been studied as regards any association between PD and labor market marginalization (LMM), and there is evidence that PD increases the risk of LMM (3, 4). There is also evidence, suggesting that especially borderline PD entails worse psychosocial function, though improvement in psychosocial function is often seen over time across all PDs (5).

There is no scientific consensus on the definition of LMM. From a social insurance perspective, LMM can be conceptualized as based on either medical assessments (resulting in sick leave or disability pension) or non-medical assessments (resulting in unemployment) (6). Studying LMM is important, as occupation, education, and income are traditional indicators of socioeconomic position (7), and there is substantial evidence showing that socioeconomic position has a major impact on health outcomes and mortality (7–9). Furthermore, there is evidence suggesting a reciprocal association between personality functioning and aspects of functional impairment, such as psychosocial functioning, indicating that interventions aimed at either domain will positively affect the other (10). This underscores the importance of studying the psychosocial effects of functional impairments in PD, such as LMM.

Some research focuses on specific areas of LMM; a few studies have been performed of the association between PD and long-term sick leave. A Norwegian study found a significant association among schizotypal, paranoid, and borderline PD and risk of long-term sick leave (11). Studies have shown ambiguous results regarding the association between PD and disability pensioning. In a Swedish register study, PD and schizophrenia/non-affective psychoses were found to bring larger increases in risk of disability pensioning and long-term sick leave than other mental disorders (3). A Norwegian study found a strong association between PD and disability pensioning in young adults. The association was stronger than for mood disorders (12). Another British study found a strong association between probable PD with comorbid mental disorders and disability pensioning, but a weak association between probable PD without comorbid mental disorders and disability pensioning (13), whereas a Finnish study found that PD increased the risk of early retirement on health grounds more than twice as much as anxiety disorders, and to an equal or slightly larger degree as depression (14). There is evidence suggesting that PD increases the risk of unemployment (4, 15). A Swedish study showed that males with PD in young adulthood had approximately 10 additional days of unemployment per year compared with peers without any mental disorder, which was comparable to the effects of other common mental disorders (4).

Another indicator of socioeconomic position is education. There is evidence suggesting an inverse relation between PD and level of education (1). An Australian study found severity of PD in young adults to be associated with not having a degree or vocational qualification and with receiving welfare benefits (16).

Although there are a number of studies of LMM and socioeconomic position in PD, there is a scarcity of studies of these outcomes where PD has been assessed reliably and using gold standard diagnostics. In 1983, Spitzer proposed a new procedure for psychiatric diagnostics: ‘longitudinal evaluation (L), done by experts (E), all (A) data (D) available’ (LEAD) (17). The LEAD procedure has been used as an index of validity in studies of psychiatric diagnostics (18, 19) and has been shown to produce stable and valid predictors of clinical status in PD over time (20). In Sweden, the LEAD procedure is considered the gold standard for psychiatric diagnostics, as the Swedish Health Technology Institute uses it for this purpose, based on a systematic literature review (21). Few studies have used a LEAD procedure in analyses of psychosocial outcomes, such as LMM, in PD.

The aim of this study was to evaluate LMM after 13 years in a clinical sample of psychiatric patients with PD reliably diagnosed in young adulthood through the LEAD procedure.

## Methods

### Participants

This study was a follow-up of a clinical cohort from 2002 to 2004 comprising all patients aged 18–25 years who sought care at a specific psychiatric out-patient clinic in Uppsala, Sweden. In the original study (22), 217 patients came for an appointment and were invited to participate, and 200 (92%) agreed. Each participant underwent a psychiatric diagnostic assessment in accordance with the LEAD procedure over three patient visits. A clinical interview was conducted by a psychiatrist during the first visit, and the Structured Clinical Interview for DSM-IV Axis I Disorders Clinical Version, SCID-I-CV (23), was conducted by the same psychiatrist during the second visit. Psychosocial problems were evaluated by a social worker during the third visit, in accordance with DSM-IV Axis IV, within nine categories (problems with the primary support group, problems related to the social environment, educational problems, occupational problems, housing problems, economic problems, problems with access to healthcare services, problems related to interaction with the legal system/crime, and other psychosocial and environmental problems). The total burden of stress was rated on a scale from 1 (none) to 6 (catastrophic) within each domain.

A team conference was held after three visits, at which all the collected information was presented, and diagnoses were established in accordance with the Diagnostic and Statistical Manual of Mental Disorders, 4th edition (DSM-IV) (24). The psychiatrists performed assessment of PDs, including the Structured Interview for DSM-IV Axis II Disorders (SCID-II) (25), for 188 participants (94%), after treatment of axis I disorder had been finalized, in order to minimize the state effect of current axis I disorders. Interrater reliability between the two psychiatrists who conducted all clinical and diagnostic interviews was measured for six randomly selected SCID-I interviews and six randomly selected SCID-II interviews (kappa coefficients of 1.0 and 0.89, respectively). The psychiatrists were both trained in accordance with the SCID manual. PD diagnosis in 188 subjects was based on observations starting at the preliminary diagnostic process (anamnesis and selected questionnaires, SCID-I, an interview with a social worker), continuing during the first treatment process, and ending during assessment of PD (SCID-II) performed by the same psychiatrist who made the initial assessments. SCID-II assesses a total of 101 symptom criteria on a scale from 1 to 3 (1 = absent, 2 = subthreshold, and 3 = threshold), resulting in a score of 101–303. Aside from symptom criteria, general PD criteria were evaluated during the interviews.

Two individuals later withdrew consent and were excluded from the study. In 2016, participants who had undergone PD diagnostics in the original study (*n* = 186) were divided into two groups based on the presence or absence of PD at baseline, yielding one group of 52 individuals who had one or more PDs at baseline (PD group) and one group of 134 individuals who had no PD at baseline (non-PD group). Within the PD group, the number of PD diagnoses per individual was 1–4: 25 individuals (48%) had one PD, 19 (10.2%) had two PDs, six (3.2%) had three PDs, and two (1%) had four PDs. The mean SCID-II score in the PD group was 164 (standard deviation [SD] 19), and that in the non-PD group was 132 (SD 15) (*t* = 10.82, *P* < 0.01). There were no significant differences in employment status or study enrollment between the two former patient groups at baseline. In the PD group, 11 individuals (21%) were employed at baseline, and 29 individuals (56%) were studying. In the non-PD group, 27 individuals (20%) were employed at baseline and 89 individuals (66%) were studying. Further characteristics of the two former patient groups are shown in Table 1. A control group was established for the study by Statistics Sweden, by matching each participant in the patient groups for sex, age, and place of residency in 2002–2003 with five control subjects (*n* = 930). Data on participants and controls were obtained from national registers from Statistics Sweden and the Swedish National Board of Health and Welfare.

**Table 1:** Description of study group at baseline 2002–2003.

Descriptive	Personality disorder, PD *n* = 52, *n* (%)	No personality disorder, non-PD *n* = 134, *n* (%)	χ^2^	*P*
**Females, *n* (%)**	38 (73)	110 (82)	0.64	0.42
**Axis I disorder**			χ^2^	*P*
Affective disorder	42 (81)	100 (75)	0.78	0.38
Anxiety disorder	42 (81)	87 (65)	4.42	0.04
Psychotic disorder	0 (0)	2 (1)	0.78	0.38
Eating disorder	17 (33)	35 (26)	0.80	0.37
Substance abuse	8 (15)	6 (4)	6.40	0.01
Other psychiatric disorder	5 (10)	8 (6)	0.77	0.38
**Number of axis I disorders**			χ^2^	*P*
0	0 (0)	6 (4)	26.50	0.001
1	7 (13)	43 (32)		
2	13 (25)	53 (40)		
3	15 (29)	19 (14)		
4	10 (19)	9 (7)		
5	7 (13)	4 (3)		
**Personality disorder**				
Paranoid	9 (17)			
Schizoid	2 (4)			
Schizotypal	1 (2)			
Borderline	15 (29)			
Histrionic	1 (2)			
Narcissistic	1 (2)			
Antisocial	4 (8)			
Avoidant	29 (56)			
Dependent	1 (2)			
Obsessive-compulsive	5 (10)			
			t	*p*
**SCID-II score, mean (SD)**	164 (19)	132 (15)	10.82	0.01
			t	*P*
**Psychosocial problems score, mean (SD)**	16 (6)	13 (4)	3.29	<0.01

SD: standard deviation; PD: personality disorders.

### Registers

Statistics Sweden is the agency responsible for holding national register data in Sweden. Statistics Sweden compiles data from various sources, such as the Swedish National Tax Agency and the Swedish Social Insurance Agency, in its registers (26, 27). This study used data from the Longitudinal integrated database for health insurance and labor market studies (LISA), withheld by Statistics Sweden. The Swedish National Board of Health and Welfare maintains national registers on Swedish healthcare and social services, such as the Swedish National Inpatient Register (IPR). The quality of parts of these registers, including the IPR, has been validated (28).

For this study, data on participants and controls for the years 2003–2016 were collected from LISA, and the Swedish National Board of Health and Welfare’s IPR register. Names and personal identity numbers of participants and controls were omitted from the retrieved register data to protect their privacy. The variables used in the study are described below and are illustrated in Supplementary Table 1.

The study was not pre-registered, and the data are not available for external use.

### Variables

#### Unemployment

Data on unemployment were collected from LISA. Data were analyzed as days of unemployment during the study period. The data collected covered days where individuals were registered as part-time unemployed, full-time unemployed, or registered in labor market measures for the unemployed.

#### Sick leave

Data on sick leave were collected from LISA. Sick leave was defined as sick leave absence >14 days, as Statistics Sweden only records information about sick leave from day 15 in LISA, due to how the Swedish social security system is devised. Sick leave absence is paid for by the employer until day 15; sick leave episodes <15 days are therefore not reported to LISA. Exceptions to this rule can be made if new sick leave episodes occur within 5 days of a previous episode. This can be registered in LISA, as it can be paid for by the Social Insurance Agency, instead of by the employer (26). Sick leave can be granted for full-time, 100%, or part-time, 75, 50, or 25%. To receive sick leave payments, an individual must have been at least partially employed during the preceding year. If there are no grounds for sick leave payment, for instance, due to unemployment, a medical certificate can mean the individual is eligible for social welfare benefits instead. In the current study, the net number of sick leave days during the study period was used in all analyses.

#### Disability pensioning

Data on disability pensioning were obtained from LISA. To receive a disability pension in Sweden, an individual’s work ability must be expected to be at least 25% reduced for at least 1 year. The work disability can be complete, 100%, or partial, 75, 50, or 25%, and can be permanent or time-limited, for example, lasting 3 years. Since 2009, it is permitted to work up to 12.5% of full-time and still retain full compensation (26). In the current study, the net number of days of disability pensioning was calculated by multiplying the degree of disability compensation with the number of days on disability pension.

#### Social welfare benefits

Data on social welfare benefits were collected from LISA. In Sweden, social welfare benefits are granted to people who are unable to financially support themselves or their children. Social welfare benefits aim to cover the regular expenses in a household. Recipients of social welfare benefits are obliged to actively search for work opportunities or take part in educational programs aiming to increase their chances of employment in the future. If an individual is not in a position to apply for work due to medical conditions but does not fulfill the criteria for sick leave payments, a medical certificate can grant the individual social welfare benefits without the requirement of applying for work (29). For this study, information was collected on the amount of money that individuals had received as social welfare benefits during the study period.

#### Income

Data on total income were collected from LISA. The variable on income which was used for analyses covered the individuals’ declared income. Social welfare benefits, sick leave payments, and study grants are not included in this register variable. Those who are self-employed can choose to declare income under a different variable.

#### Education

Data on the highest attained level of education at age 30 years were collected from LISA. Data were dichotomized into two groups: up to secondary education (≤12 years) and post-secondary education (>12 years).

#### Disease burden due to psychiatric disorders

Days admitted to hospital for psychiatric care were calculated using data from the IPR. The IPR records day of admittance and day of discharge for all admittances to hospitals in Sweden. Admittances recorded in the IPR are assigned a code unique to the admitting department (e.g. psychiatry, geriatrics, and pediatrics). The IPR also links each admittance to diagnoses in the International Classification of Diseases (ICD) (28). For this study, days registered as being in care at departments with an IPR code referring to psychiatric care were used.

#### Missing data

Data were missing for participants who had resided abroad at any time during the study period. Therefore, these participants were excluded, as well as their matched controls. Participants who had died during the study period were also excluded from analyses, as well as their matched controls. Other controls who had resided abroad or had died during the study period were also excluded.

### Statistics

Chi-squared tests were performed for proportional differences between the study groups. When comparing the two study groups with the control group, the Wald chi-square test was used.

IBM SPSS Statistics for Macintosh, version 28.0, was used for all computations. The level of significance was set at 5%.

### Ethics approval

This study was approved by the Regional Ethics Committee in Uppsala (Dnr 2017/251).

## Results

The characteristics of the two former patient groups are shown in Table 1. There were significant differences between these groups regarding prevalence of anxiety disorders and substance abuse. The PD group had significantly more comorbid axis I disorders and a higher mean psychosocial problem score at baseline than the non-PD group. The PD group also had a significantly higher mean SCID-II score.

Five individuals (9.6%) were excluded from the PD group because of death or residency abroad during the study period. The corresponding figure in the non-PD group was 13 (9.7%). Their matching controls (*n* = 90) were excluded. Furthermore, there were 86 controls who had resided abroad or died during the study period, who were also excluded.

### Labor market outcomes and receipt of psychiatric care

Days of unemployment, sick leave, and disability pensioning were added up and merged into the new variable, LMM. The mean number of days in LMM for the PD group was 838 (95% confidence interval [CI] 627; 1,121), that for the non-PD group was 665 (95% CI 502; 881), and that for the control group was 440 (95% CI 380; 508). At age 30 years, 76.1% of the PD group had attained an education beyond high school (>12 years). The corresponding figure in the non-PD group was 85.1%, and that in the control group was 68.2%. During the study period, 44.7% of the PD group had ever been admitted to psychiatric care. The corresponding figure was 22.3% for the non-PD group and 3.7% for the control group. The PD group had a mean yearly income at age 30 years of 173 (× 100 US dollars) (95% CI 131; 227). The non-PD group had a mean yearly income at age 30 years of 219 (× 100 US dollars) (95% CI 191; 251), and controls 236 (× 100 US dollars) (95% CI 225; 248). In the PD group, 42.6% of individuals had received social welfare benefits at some point during the study period. The corresponding figure for the non-PD group was 23.1%, and that for controls was 13%. For the PD group, the mean amount of social welfare benefits received during the study period was 92 (× 100 US dollars) (95% CI 47; 182), that for the non-PD group was 17 (× 100 US dollars) (95% CI 8; 34), and that for controls was 14 (× 100 US dollars) (95% CI 10; 21).

**Figure 1:**
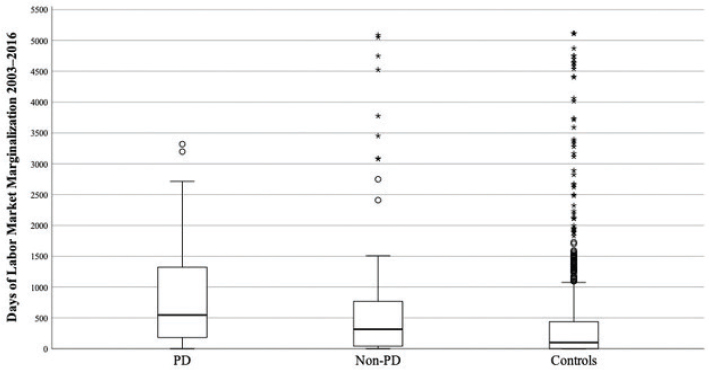
Days of labor market marginalization (unemployment, sick leave, and disability pensioning) in 2003–2016 in the groups PD, non-PD, and controls. PD: personality disorder.

When comparing the two study groups (PD and non-PD) with controls, significant differences were found in regards days in LMM, ever receipt of social welfare benefits, and ever admittance to psychiatric care. When comparing the two study groups with each other, the PD group was found to have received significantly more social welfare benefits during the study period. The PD group also had significantly more participants who had ever been admitted to psychiatric care during the study period compared with the non-PD group. The PD group had a lower income at age 30 years compared with the non-PD group and controls, but the finding was only significant compared with controls. Both study groups had a higher level of education at age 30 years compared with controls, but this finding was only significant for the non-PD group. The results are shown in Figure 1 and Table 2.

**Table 2:** Labor market outcomes, educational level, and receipt of psychiatric care 2003–2016 for the groups PD, non-PD, and controls.

Outcome	PD group *n* = 47 mean (SD)	Non-PD group *n* = 121 mean (SD)	Controls *n* = 754 mean (SD)	Wald χ^2^	Post hoc test
Days in LMM in 2003–2016 (95% CI)	838 (627; 1,121)	665 (502; 881)	440 (380; 508)	18.3	PD > controls** Non-PD > controls*
Amount of social welfare benefits received in 2003–2016 (95% CI) (× 100 US dollars)^Ç^	92 (47; 182)	17 (8; 34)	14 (10; 21)	26.9	PD > Non-PD* PD > Controls*
Income at age 30 years (95% CI) (× 100 US dollars/year) ^Ç^	173 (131; 227)	219 (191; 251)	236 (225; 248)	6.3	PD < Controls*
	*n* (%)	*n* (%)	*n* (%)	Wald χ^2^	*Post hoc test*
Ever receipt of social welfare benefits in 2003–2016 (%)	20 (42.6)	28 (23.1)	98 (13.0)	29.4	PD > Non-PD* PD > Controls** Non-PD > Controls**
Education > 12 years at age 30 years, *n* (%)	35 (76.1)^a^	103 (85.1)	514 (68.2)	14.0	Non-PD > Controls***
Ever admitted to psychiatric care, *n* (%)	21 (44.7)	27 (22.3)	28 (3.7)	98.6	PD > Non-PD*** Non-PD > Controls** PD > Controls***

SD: standard deviation; PD: personality disorder; CI: confidence interval; LMM: labor market marginalization.

* *P* < 0.05,

** *P* < 0.01,

*** *P* < 0.001.

^Ç^Converted from Swedish kronor (SEK) to US dollars (USD) using an exchange rate of 1 SEK = 0.1005 USD, based on data from May 2022.

a *n* = 46 due to incomplete register data.

## Discussion

The results indicated that mental disorder in young adulthood increased LMM up to 13 years after diagnosis, with the two study groups having more days of LMM and more often having received social welfare benefits compared with controls. The significant differences in LMM in the study were mainly between former patients (PD group and non-PD group) and controls, though the former patient PD group showed more distinct differences regarding the receipt of social welfare benefits. The PD group also had a lower level of yearly income at age 30 years than the other groups. There could be several reasons for these findings. First, there was a certain degree of overlap in PD symptoms between the two former patient groups when the data were approached with a dimensional view of PD. Also, both former patient groups were heavily burdened with axis I comorbidity at baseline. Although the PD group had significantly more axis I disorders, the long-term outcomes in the non-PD group were most likely negatively affected by this burden. It is striking that the former patient groups had worse outcomes than controls, although both groups had more individuals with education >12 years at age 30 years than the controls. Age 30 years was chosen as point of reference in a similar study by Jonsson et al. (30). It would be of interest to compare participants with controls later on in life.

It would have been of interest to account for social covariates at baseline, for example, parental socioeconomic situation. Upon selecting data to be retrieved from Statistics Sweden and the National Board of Health and Welfare, there was a concern that more individual data would risk breaches to anonymity of participants. We therefore refrained from gaining information on some factors from baseline which would have been of interest to account for, such as country of birth. Sweden is a country with relatively small economic gaps, where 45% of the population has higher education (31), and it could therefore be assumed that no great differences in parental socioeconomic situation existed between groups at baseline.

Another issue to address when interpreting results is the effect that outliers had on mean values. As Figure 1 shows, the mean value in the non-PD group might be increased because of the outliers with extremely high LMM values. These extreme values were also present in the control group. However, as the control group was much larger (*n* = 930), the effect of extreme values would be lower there. We do not know the reasons for these extreme LMM values, but they may have been caused by somatic disease, as they were also present in the control group.

Avoidant PD was the most common PD in the study. Anxiety disorders, such as social phobia, were significantly more common in the PD group than in the non-PD group. The Diagnostic and Statistical Manual of Mental Disorders, 5th edition (DSM-5) (32), describes an overlap between avoidant PD and social phobia, and that the two diagnoses may stem from the same or similar personality trait. In the current study, PD diagnoses were assessed after finalization of treatment of axis I disorders. This decreased the risk of misdiagnosis between axis I and II, but there was a risk that individuals actually suffering from avoidant PD were diagnosed with social phobia, which would have affected results. Also, comorbidity between PD and axis I disorder is common (33). It is possible that the higher degree of axis I comorbidity in the PD group would account for the findings. In the current study, it was not possible to use axis I disorders as a covariate in the analyses, due to the characteristics of the register data used.

Our findings are in line with those of previous studies of LMM in PD (1, 3, 4, 11, 12, 14, 15). This study adds further validity to previous findings, thanks to the use of gold standard diagnostics based on the LEAD procedure.

The findings are important for various reasons. First, there is a scientific discourse on the validity of PD diagnoses (34). The findings in the current study indicate validity of PD diagnostics in young adulthood, as worse outcomes in psychosocial functioning were found in the PD group over time. Second, LMM as an aspect of socioeconomic position is important for many health outcomes and may in itself lead to worsened mental health (35–37).

Individuals in the PD group received a mean amount of 9,208 US dollars in social welfare benefits during the study period (corresponding to on average 658 US dollars per year), which was significantly more than the other groups. The PD group had a lower yearly income at age 30 years than other groups. The variable on income which was used for analyses covered the individuals’ declared income; however, social welfare benefits, sick leave payments, and study grants were not included in this register variable. Furthermore, individuals who are self-employed can choose to declare income in a manner which will become registered under a different variable in LISA. We therefore ran analysis of the other variable (KuINK) at age 30 years for those who had been registered as self-employed in LISA. The difference in results for these individuals when comparing the declared income variable with income declared as the variable KuINK was minimal. In Sweden, future pension is largely based on income during working years, meaning lower income results in lower future pension payments. The worse labor market outcomes in the PD group might therefore have life-long impacts. The pattern of increased LMM found in the current study among those with PD calls for urgency in addressing and, when possible, treating PD.

This study did not look into why PD affects LMM. Previous studies have found that PD is associated with dysfunctional behaviors at work, leading to adverse work outcomes such as being fired, laid off, or experiencing problems in interactions with coworkers and bosses (15). Further studies of this are suggested.

There are a number of limitations to the study. First, it is problematic that the PD group was assessed as a single entity, as PD diagnoses differ in areas of functional impairment; something which is easy for an individual with a certain PD can be difficult for an individual with another PD. Comorbidity between PDs as defined in the DSM-IV (24) is common (34). Occurrence of more than one PD in the same individual is often interpreted by clinicians as an indication of severe PD. In the DSM-5 (32), Section III, a dimensional approach to PDs is suggested as an alternative model of PD (38). This model has been suggested to be useful for the study of psychosocial functioning in PD, as it places a general transdiagnostic personality dysfunction severity factor at the core of the PD entity (2). In the current study, the dichotomic PD construct was used primarily, but an attempt at a more dimensional approach to the PD construct was adopted by analyzing the SCID-II scores and comparing these between the two former patient groups. The two groups differed significantly in total SCID-II scores, but there was a certain degree of overlapping scores. It would have been of interest to analyze outcomes based on a dimensional PD approach through the SCID-II score instead of the dichotomized PD categories. However, this was not possible due to the characteristics of the retrieved register data.

Another limitation to the current study was the small sample size, especially in the PD group (*n* = 52). Small effect sizes in such small groups lead to non-significant results, making it hard to draw conclusions. However, using the LEAD procedure for PD produces valid and reliable diagnoses. The two former patient groups differed significantly in mean SCID-II scores, increasing the reliability of findings. It was within the scope of the study to use this strength to make up for the low power created by the small sample size.

There are many strengths to using Swedish national registers for data collection. Some of the registers have been validated (28), and they are generally considered to have high quality. However, the registers are not created for scientific purposes and reflect changes in politics and society in Sweden over time. This has resulted in many changes to the variables over time, which sometimes complicates the study of register data over longer periods of time. One could argue that merging sick leave, disability pensioning, and unemployment into a compound LMM variable, instead of studying each variable on its own, limits the interpretability of findings. However, the compound LMM variable reflects lowered vocational functioning and ability of financial self-support, irrespective of the underlying reason, which was a main aim of the study. A compound LMM variable has been used in previous Swedish long-term studies of psychosocial outcomes in psychiatric disorders (39).

Another weakness of the study concerns the quality of the specific data collected from the registers. Days of sick leave, disability pensioning, and unemployment were merged into the new variable LMM and analyzed together. However, social welfare benefits were analyzed separately, as there were no data on the number of days that social welfare benefits had been received, only on the amount of money received. It would have been useful to have access to data on the number of days with social welfare benefits, to enable comparison with collected data on LMM, but such data were not available. There was also a limitation in the data collection of variables regarding disability pension. The registers include information on the net number of days on disability pension. However, this was overlooked when collecting the data, with the gross number of days being collected instead. The net number of days was calculated by multiplying the gross number of days with the fraction of received disability pension. This yielded a margin of error, as data on gross number of days and fraction of received sickness pension are compiled by Statistics Sweden from different data sources. The error was small and is not likely to have had any significant impact on the findings.

The number of days admitted to hospital for psychiatric care was used to reflect the disease burden of psychiatric disorders other than PD in this study. This could also have been studied using the ICD code that admittances were assigned in the IPR. However, there would have been several limitations to that strategy. First, the risk of invalid diagnoses being made by clinicians during admittance would have had to be considered. Second, it is more common in psychiatric care than in somatic care to have missing primary diagnoses in the IPR after the year 2000 (28).

Another limitation of the study is that the control group was matched for sex, age, and living location at study baseline. However, the occurrence of PD in the control group is not known. The prevalence of PD in the general population is disputed, with studies suggesting it to be 9–12% (40, 41). The control group for the current study was chosen to match participants and, hence, is not representative of the general population. It is therefore difficult to estimate the proportion of PD in the control group. When interpreting the results, it should be considered that a certain proportion of individuals in the control group are likely to have fulfilled diagnostic criteria for PD, which could have given this subgroup similar results to those of the PD group. This would have had an attenuating effect on the results.

In conclusion, in this study, former psychiatric patients with and without PD diagnosed in young adulthood, followed up after 13 years, had more days of LMM and received more social welfare benefits than controls. Findings were mostly significant between former patient groups and controls, but the PD group stood out as regards receipt of social welfare benefits. Results suggest that PD diagnosed in young adulthood impairs social functioning in the long term.

## Data Availability

The analyze code can be made available upon request. Data will not be made publicly available but will be made available upon reasonable request.
