# Supplementary material for: A long-term follow-up study of labor market marginalization in psychiatric patients with and without personality disorder
Source: Ups J Med Sci. 2023 Jul 31;128:10.48101/ujms.v128.9014. doi: 10.48101/ujms.v128.9014 (PMC10392854; doi:10.48101/ujms.v128.9014)
Supplement: Supplementary file 1 [file UJMS-128-9014-s001.pdf]

Supplementary table S1

Name of variables in the study, included register variables in study variables, and source of the respective variables.

**Name of variable in study**	**Description**	**Register variables**	**Source**
Labor market marginalization (LMM)	Unemployment	AdelDag	LISA
		AK14Dag	
		ALosDag	
		AstuDag	
		ASysDag	
	Disability pensioning	AktErs_Bdag_MIDAS	LISA
		Sjukers_Bdag_MIDAS	
		Rehab_Bdag_MIDAS	
		Rehab_Forlängd_Bdag	
		SjukErsGarAnd	
		SjukErsInkAnd	
		AktErsGarAnd	
		AktErsInkAnd	
	Sick Leave	SjukSum_Ndag_MIDAS	
Education at age 30 years	Academic degree	Sun2000niva	LISA
Social welfare benefits	Amount of social welfare benefits received during study period	Socbidrpersf	LISA
Income at age 30 years	Declared income at age 30 years	DekLon	LISA

Days of psychiatric care	Days admitted to psychiatric inpatient care during study period	MVO901	National Inpatient register (IPR)
		MVO906	
		MVO928	
		MVO931	
		MVO943	
		MVO944	
		MVO945	
		MVO953	
		MVO954	
		MVO955	
		MVO956	
		MVO957	
		MVO991	
		MVO993	
